# Supplementary material for: A versatile marine modelling tool applied to arctic, temperate and tropical waters
Source: PLoS One. 2020 Apr 10;15(4):e0231193. doi: 10.1371/journal.pone.0231193 (PMC7147738; doi:10.1371/journal.pone.0231193)
Supplement: S1 File — (DOCX) [file pone.0231193.s001.docx]

## FlexSem setup example

Example of input file to the precompiled FlexSem program. A very simple 0D box model that simulates nutrient limited phytoplankton growth. The example can be run in the command prompt by calling FlexSem.exe with the setup file as input parameter:

FlexSem.exe eco0.xml

This will generate a log file (eco0.log) and the output file defined in the setup (eco0.txt). The selected output format (EMF) is a fixed width text format, which can easily be read and plotted in R.

### Setup file (eco0.xml)

<EM>

<General>

<Mesh>

<Filename>onebox.mesh</Filename>

</Mesh>

<Bathymetry>1</Bathymetry>

<LayerThickness>1</LayerThickness>

<StartDT>2010-01-01</StartDT>

<DT>1</DT>

<NoTimesteps>100</NoTimesteps>

<LogLevel>10</LogLevel>

</General>

<Constants>

<Constant><Symbol>mortality</Symbol><Value>0.2</Value></Constant>

<Constant><Symbol>maxGrowthRate</Symbol><Value>20</Value></Constant>

<Constant><Symbol>halfSat</Symbol><Value>100</Value></Constant>

</Constants>

<FlexSem>

<Variables>

<Variable><Symbol>uptake</Symbol><InitValue>0</InitValue></Variable>

<Variable><Symbol>nut</Symbol><InitValue>100</InitValue><NotNegative>true</NotNegative></Variable>

<Variable><Symbol>phyto</Symbol><InitValue>0</InitValue></Variable>

</Variables>

<Equations>

<Equation><Definition>uptake = maxGrowthRate*(nut/(nut+halfSat))</Definition></Equation>

<Equation><Definition>nut = nut - uptake</Definition></Equation>

<Equation><Definition>phyto = phyto + uptake - (mortality*phyto)</Definition></Equation>

</Equations>

</FlexSem>

<Outputs>

<Output>

<Filename>eco0fs.txt</Filename>

<Format>EMF</Format>

<Variable>phyto</Variable>

<Variable>nut</Variable>

<Variable>uptake</Variable>

</Output>

</Outputs>

</EM>

### Mesh file (onebox.mesh)

4 1

0 0

1 0

1 1

0 1

0 1 2 3

### R script to read and plot the data

d <- read.fwf("eco0fs.txt",c(19,6,12,12,12),skip=1)

matplot(d[,3:4],xaxt="n",ylab="N",xlab="Time",type="l")

legend("topright",c("Phytoplankton","Nutrients"),lty=c(1,2),col=c("black","red"))

**S1 Fig. Model output.**
