# Supplementary figures and images for: A versatile marine modelling tool applied to arctic, temperate and tropical waters
Source: PLoS One. 2020 Apr 10;15(4):e0231193. doi: 10.1371/journal.pone.0231193 (PMC7147738; doi:10.1371/journal.pone.0231193)

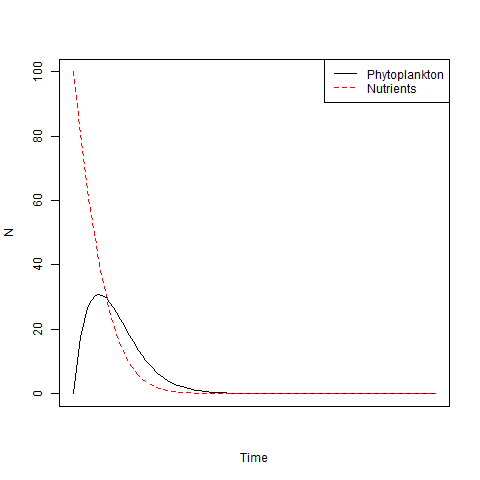

Supplement: S1 Fig — (PNG) [file pone.0231193.s002.png]
